# Impact of Media-Based Age Stereotypes on Older Individuals’ Mental Health During the COVID-19 Pandemic

**DOI:** 10.1093/geroni/igab046.2264

**Published:** 2021-12-17

**Authors:** E-Shien Chang, Sarah Lowe, Natalia Provolo, Martin Slade, Becca Levy

**Affiliations:** 1 Weill Cornell Medicine, New York City, New York, United States; 2 Yale University, New Haven, Connecticut, United States; 3 Yale University, Woodbridge, Connecticut, United States

## Abstract

During the COVID-19 pandemic, stigmatization of older persons has increased in traditional and social media. It was unknown whether this negative messaging could be detrimental to the mental health of older individuals, and whether the relatively uncommon positive messaging about older individuals could benefit their mental health. To address these gaps, we designed age-stereotype interventions based on actual news stories that appeared during the pandemic. As expected, the exposure of older individuals to the negative-age-stereotype-messaging interventions led to significantly worse mental health (more anxiety and less peacefulness), compared to a neutral condition; in contrast, the positive-age-stereotype-messaging interventions led to significantly better mental health (less anxiety and more peacefulness), compared to a neutral condition. The results demonstrate the need for media messaging aimed at empowering older individuals during the pandemic and beyond.

